# Subclinical Pregnancy Toxemia-Induced Gene Expression Changes in Ovine Placenta and Uterus

**DOI:** 10.3389/fvets.2016.00069

**Published:** 2016-08-30

**Authors:** Ramanathan K. Kasimanickam

**Affiliations:** ^1^Department of Veterinary Clinical Sciences, College of Veterinary Medicine, Washington State University, Pullman, WA, USA

**Keywords:** sheep, pregnancy toxemia, uterus, placenta, gene expression

## Abstract

The objective was to elucidate gene expression differences in uterus, caruncle, and cotyledon of ewes with subclinical pregnancy toxemia (SCPT) and healthy ewes, and to identify associated biological functions and pathways involved in pregnancy toxemia. On Day 136 (±1 day) post-breeding, ewes (*n* = 18) had body condition score (BCS; 1–5; 1, emaciated; 5, obese) assessed, and blood samples were collected for plasma glucose and β-hydroxybutyrate (BHBA) analyses. The ewes were euthanized, and tissue samples were collected from the gravid uterus and placentomes. Based on BCS (2.0 ± 0.02), glucose (2.4 ± 0.33), and BHBA (0.97 ± 0.06) concentrations, ewes (*n* = 10) were grouped as healthy (*n* = 5) and subclinical SCPT (*n* = 5) ewes. The mRNA expressions were determined by quantitative PCR method, and prediction of miRNA partners and target genes for the predicted miRNA were identified using miRDB (http://mirdb.org/miRDB/). Top ranked target genes were used to identify associated biological functions and pathways in response to SPCT using PANTHER. The angiogenesis genes *VEGF* and *PlGF*, and *AdipoQ, AdipoR2, PPARG, LEP, IGF1, IGF2, IL1b*, and *TNF*α mRNA expressions were lower in abundances, whereas hypoxia genes *eNOS, HIF1a*, and *HIF 2a*, and *sFlt1* and *KDR* mRNA expressions were greater in abundances in uterus and placenta of SCPT ewes compared to healthy ewes (*P* < 0.05). The predicted miRNA and associated target genes contributed to several biological processes, including apoptosis, biological adhesion, biological regulation, cellular component biogenesis, cellular process, developmental process, immune system process, localization, metabolic process, multicellular organismal process, reproduction, and response to stimulus. The target genes were involved in several pathways including angiogenesis, cytoskeletal regulation, hypoxia response *via* HIF activation, interleukin signaling, ubiquitin proteasome, and *VEGF* signaling pathway. In conclusion, genes associated with blood vessel remodeling were lower in abundances and that the genes associated with hypoxic conditions were greater in abundances in the uteroplacental compartment of SCPT ewes. It is obvious that the factors that influence placental vascular development and angiogenesis as noted in this study set the course for hemodynamic changes and hence have a major impact on the rate of transplacental nutrient exchange, fetal growth, and health of the dam.

## Introduction

Pregnancy toxemia is a metabolic disorder of pregnant ewes, caused by an abnormal metabolism of carbohydrates and fats, which occurs during the final stage of pregnancy. The disease occurs more frequently in lean [body condition score (BCS) <2 in the 5-point scale] or obese (BCS ≥4) animals, as well as in animals carrying two or more fetuses (1–4).

In ewes, glucose is the principal carbon source for placental and fetal oxidative metabolism and tissue formation (5, 6). A total of 30–50% of maternal glucose production in late gestation is taken up by uterine and fetal tissues (5–8), and 50–70% of this amount is used by the uteroplacental unit (6, 9, 10). During late gestation, increased energy demands of the rapidly developing fetus(es) cause an unbalanced lipid and carbohydrate metabolism in the pregnant animal and putting them at risk to pregnancy toxemia (1, 2). During late pregnancy, the impaired fat and carbohydrate metabolism produces increased levels of fatty acids and ketone bodies, mainly β-hydroxybutyrate (BHBA), besides the decreased glucose concentration (3, 4).

Pregnancy requires an expansion of maternal blood volume, an increase of cardiac output, and a redistribution of blood to the uterus to meet the needs of the growing fetus. Normal maternal vascular adaptation during pregnancy includes enhanced vasodilation, with the greatest effect seen in the uterus. A range of pathophysiological factors including maternal stress due to poor nutrition, hyperthermia, or metabolic diseases such as pregnancy toxemia and eclampsia may affect important metabolic, transport, and hemodynamic functions of placentas. Excessive accumulation of free radicals affects placental development and function, and may subsequently impact both the fetus and dam (11–14).

Hypoxia plays critical roles in vascular development during embryonic and fetal growth *in utero* (15). In endothelial cells, hypoxic conditions drive the transcription of multiple genes, which control vascular function, expansion, and remodeling. Although tissue hypoxia is the main driving force for angiogenesis, a growing body of evidence has demonstrated that oxidative stress can also be a potent trigger for the development of new vessels. However, high level of acute oxidative stress and/or chronic oxidative stress has a vital role in development of vascular diseases (16), including placental ischemia. Development of insulin resistance diabetes and cardiovascular disease were associated with increased oxidative stress. Insulin resistance has been implicated as causative factor in the pathogenesis of ovine pregnancy toxemia (17).

The objective of this study was to determine the changes in gene expressions in uterus, caruncle, and cotyledon of ewes with subclinical pregnancy toxemia (SCPT).

## Materials and Methods

### Animals and Sample Collection

Blood and tissue samples were collected during a previously reported clinical trial to determine the effect of daily tocopherol supplementation during late stage of pregnancy. Briefly, 18 pregnant ewes (3.1 ± 0.11 years of age; Dorset cross; impregnated by two different sires by natural service) with similar breeding dates were selected and were maintained under normal pasture conditions. One week prior to the trial, the selected ewes were moved to the research facility. The ewes had access to 35 sq. ft/ewe paddock lots. In addition, they were fed free choice hay. The ewes were randomly assigned to receive (1) 500 mg of α-tocopherol (*n* = 6), (2) 1,000 mg of γ-tocopherol (*n* = 7), or (3) no treatment (*n* = 5) received a placebo. Animals were supplemented orally, once daily, from approximately 100 to 136 (±1) days post-breeding. On Day 136 (±1) post-breeding, all ewes received BCS (1–5; 1, emaciated; 5, obese), and blood samples were collected by jugular venipuncture for plasma glucose and BHBA (in tubes anticoagulated with heparin 10 U/mL) and serum 8-isoprostane (in tubes without heparin) determination. All ewes were euthanized (Day 136 ± 1), and tissue samples were collected immediately from the gravid uterus (full thickness) and placentomes (caruncle and cotyledon). Placentome and uterine samples were collected close to the umbilicus for consistency. Cotyledons were separated from caruncles by applying strong pressure. Tissue samples were placed in RNAlater (Qiagen Inc., Valencia, CA, USA) in 5-mL Nalgene^®^ cryogenic vials (Sigma-Aldrich, St. Louis, MO, USA) and snap frozen immediately and stored at −70°C for subsequent evaluation of mRNA expression. This study was approved by Virginia Tech Institutional Animal Care and Use Committee (IACUC; 04-068-CVM). Tissue Use Protocol was approved by IACUC at Washington State University (ASAF #03922-001).

### Determination of Glucose, β-Hydroxybutyrate, and Isoprostane

Blood samples (with heparin) were centrifuged at 3,500 × *g* for 15 min, and plasma was separated and stored at −20°C until analyzed. Determination of plasma glucose was done by the glucose oxidase enzymatic method (MyBioSource, LLC, San Diego, CA, USA) as described in the previous reports (18, 19), and the BHBA was measured enzymatically as described previously (20), in triplicates using 96-well plates. Plates were read using Glomax^®^-Multi Detection System (Promega Corporation, Madison, WI, USA).

Blood samples (without heparin) were centrifuged at 1,200 × *g* for 10 min, and serum was separated and stored at −20°C until analyzed. Isoprostane in serum samples were estimated by direct ELISA as described previously (21). Briefly, 100 μL of anti-goat-8-epi-PGF2α antibody (MyBioSource, LLC, San Diego, CA, USA) was added in the 96-well plates that were pre-coated with standard or samples and kept at 4°C for at least 24 h. After washing with buffer, 100 μL of secondary antibody, raised in donkey anti-goat IgG-HRP (Santa Cruz Biotechnology, Inc.), was added to each well. After washing with buffer, 200 μL of reagent containing the substrate of acetyl cholinesterase and then 50 μL of stop solution were added. Plates were read at 450 nm using Glomax^®^-Multi Detection System (Promega Corporation, Madison, WI, USA), and serum concentrations of isoprostane were calculated from standard curves.

### Real-Time Polymerase Chain Reaction

#### Total RNA Extraction from Tissues

Total RNA was extracted from uterus, caruncle, and cotyledon tissues with RNeasy Mini Kit (QIAGEN Inc., Valencia, CA, USA) according to the manufacturer’s protocol. RNA concentration was measured using a NanoDrop spectrophotometer (Thermo Fisher Scientific Inc., West Palm Beach, FL, USA). Sample absorbance ratio of 260/280 wavelength was observed to ensure the purity of RNA and they were 1.96–2.00. DNase treatment was performed using deoxyribonuclease 1 (amplification grade, Invitrogen™, Carlsbad, CA, USA). Briefly, the 1 μg of RNA sample was added with 1 μl of 10× DNase I reaction buffer, 1 μl of 10 DNase I enzyme, and DEPC-treated water. The mix was incubated for 15 min at room temperature. After the reaction, the enzyme was inactivated by adding 1 μl of 25 mM EDTA and heating to 65°C for 10 min. Then, the RNA samples were stored at −20°C until complementary DNA (cDNA) preparation.

#### Polymerase Chain Reaction of Selected Genes of Interest

The mRNA was reverse transcribed to cDNA. The cDNA samples were prepared using the iScript cDNA Synthesis kit (Bio-Rad Laboratories, Hercules, CA, USA). A 500-ηg sample of RNA was reverse transcribed in 20-μL reaction at the incubating conditions of 25°C for 5 min, 42°C for 30 min, and 85°C for 5 min; 25 ηg/μL RNA equivalent cDNA was obtained. Qiagen Tag PCR master mix (Qiagen, Valencia, CA, USA), a pre-mixed solution, was used to amplify the fragment of the genes of interest. Final concentration of the primers was 0.3 μM. Initial denaturation was set at 94°C for 3 min. Followed by 30 cycles of denaturation at 94°C for 1 min, annealing at 55°C for 1 min and extension at 72°C were programed. A final extension step at 72°C for 10 min was included in thermocycling conditions. Primers (Table S1 in Supplementary Material) were designed either using the NCBI website or primer express version 3.0 (Applied Biosystems Inc., Carlsbad, CA, USA). Consideration was given to the set of primers (forward and reverse primers) to ensure separation of at least an intron and melting temperatures, and CG content were set at optimal, or close to optimal level. Amplicon was run on a 2% agarose gel and stained with ethidium bromide for visualization to ensure a single amplicon for a set of primers.

#### Determination of mRNA Expression Using Real-Time PCR

SYBR green chemistry was applied to observe relative mRNA expression. Fast SYBR green master mix (2×) (Applied Biosystems Inc., Carlsbad, CA, USA) was used to prepare the reaction mix. The final concentration of each primer was 0.3 μM. A 20-μL aliquot of three technical replicates was used for each sample. A 1.6-μL volume of 25 ng/μL RNA equivalent cDNA was present in the total volume of the three triplicates. StepOne Plus instrument (Applied Biosystems Inc., Carlsbad, CA, USA) was used for the real-time PCR runs. Precycling stage was maintained at 95°C for 20 s. Forty cycles of amplification was carried out with the conditions of 95°C for 3 s and 60°C for 30 s (fast ramp speed conditions for the fast mixture). A continuous dissociation step was added to look for additional amplification products.

Carboxy-X-rhodamine (ROX) dye was set up for the passive internal reference. The baseline was automatically adjusted to obtain threshold cycles of each sample. Threshold cycles were normalized to an endogenous control, β-actin. A standard curve was obtained using one in five dilutions for each set of primer in order to check the amplification efficiency. Correlation coefficient for the dilution curve was ≥0.9900.

### Morphometry Analysis of Placental Unit

The placentome samples collected for histological evaluation were fixed in 10% neutral buffered formalin, sectioned at 5 μm, and stained with hematoxylin and eosin. They were evaluated on a Nikon E400 Eclipse microscope, and photomicrographs (100×) were taken with a Nikon camera with a 3 chip. Images were processed with Nikon Act 1 software. Image processing and morphometry analysis were performed using ImageJ 1.42q (NIH, USA) to evaluate the fractal dimension and lacunarity as described previously (22). A fractal dimension is a scaling rule comparing how a pattern’s detail changes with the scale at which it is considered. The fractal dimension is a valuable parameter to describe the complexity. Lacunarity is a measure of homogeneity of structure or the degree of structural variance within an object. Briefly, FarcLac 2.5 (NIH, USA) was used to perform fractal dimension. The FracLac scan images using a shifting grid algorithm that can do multiple scans from different locations on each image. The average value over all locations was considered as the final estimate of fractal dimension. During the same analytical process, lacunarity was also calculated. It was estimated as the average of the coefficient of variation for pixel density over all grid sizes and locations. A total of 30 locations were evaluated for each sample.

### Prediction of Functional Gene and miRNA Partners, and Its Biological Function and Pathways

Predictions of functional gene network and their miRNA partners were determined using GeneMANIA prediction server, as described previously (23–26). Target genes were predicted using miRDB (http://mirdb.org/miRDB/) for the predicted miRNAs, and top ranked predicted genes were run using PANTHER classification system (27) to identify associated biological processes and pathways in response to pregnancy toxemia.

### Animal Grouping and Data Management

Based on BCS, glucose, and BHBA concentrations (Table 1), ewes (*n* = 10) were grouped as healthy (*n* = 5) and SCPT (*n* = 5) ewes (4). In the previous studies (21, 22, 28), tocopherol treatment was considered as the main effect. The alpha and gamma tocopherol concentrations in placenta, uterus, and serum on Day 136 between healthy and SCPT ewes were not found to be different (Table S2 in Supplementary Material). Thus, tocopherol treatment categories were excluded in the overall analysis of this study. Ewes’ serum isoprostane concentrations, mRNA relative fold changes and values of fractal dimension, and lacunarity of placental unit were grouped for the healthy and the SCPT categories for analysis. Three ewes in SCPT group had twins.

**Table 1:** **Mean ± SEM body condition scores (BCSs) and plasma glucose, and β-hydroxybutyrate (BHBA) concentrations in healthy ewes (*n* = 5) and ewes with subclinical pregnancy toxemia (*n* = 5)**.

BCS		Glucose (mmol/L)		BHBA (mmol/L)	
Healthy ewes	SCPT ewes	Healthy ewes	SCPT ewes	Healthy ewes	SCPT ewes
2.6 ± 0.05^a^	2.0 ± 0.02^b^	3.2 ± 0.10^a^	2.4 ± 0.33^b^	0.34 ± 0.05^a^	0.97 ± 0.06^b^

*SCPT, subclinical pregnancy toxemia*.

### Statistical Analysis

The data were analyzed using SAS software (SAS version 9.12, SAS Institute Inc., Cary, NC, USA). Mean ± SEM differences in BCSs and plasma glucose and BHBA concentrations between healthy and SCPT ewes were calculated using ANOVA. Mean ± SEM differences in alpha tocopherol and gamma tocopherol concentrations in placenta, uterus, and serum of healthy SCPT ewes were calculated using ANOVA. The differences in fractal dimension and lacunarity in uteroplacental unit between healthy and SCPT ewes were tested by ANOVA. The PCR data were subjected to ANOVA using 2^−ΔΔCt^ values to ascertain statistical significance of any differences in genes expressions between healthy and SCPT ewes (29).

## Results

### Plasma Glucose and β-Hydroxybutyrate, and Serum Isoprostane Concentrations

Mean ± SEM BCS and plasma glucose, and BHBA concentrations for healthy ewes (*n* = 5) and for ewes with SPCT (*n* = 5) are presented in Table 1. The BCS and concentrations of glucose were lower and BHBA was greater in SCPT ewes compared to healthy ewes (*P* < 0.05). The serum isoprostane concentrations were different between healthy and SCPT ewes, 244.6 ± 14.2 and 292.9 ± 9.1, respectively (*P* < 0.05).

### mRNA Expression between Healthy and Subclinical Pregnancy Toxemia Ewes

**Figure 1:**
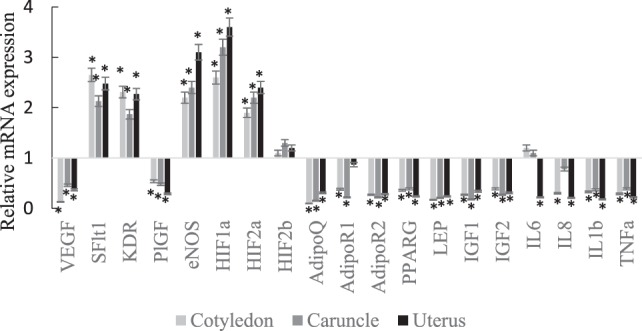
**mRNA expression (±SEM) of vascular endothelial growth factor (*VEGF*), kinase insert domain receptor (*KDR*), soluble Fms-like tyrosine kniase-1 (*sFlt1*), endothelial nitric oxide synthase (*eNOS*), and placental growth factor (*PlGF*), hypoxia-inducible factors (*HIF-1a, HIF-2a*, and *HIF-2b*), adiponectin (*AdipoQ*), adiponectin receptor (*AdipoR*)*1, AdipoR2*, peroxisome proliferator-activated receptor gamma (*PPAR*γ), insulin-like growth factor (*IGF*)*1, IGF2*, and leptin (*LEP*), interleukin (*IL*)*-6, IL-8, IL-1b*, and tumor necrosis factor alpha (*TNF*α)**. (Gene expression of the control was set at 1, and gene expression of SGPT was related to the control; *P < 0.05).

The *VEGF, PlGF, AdipoQ, AdipoR2, PPARG, Lep, IGF1, IGF2, IL1b*, and *TNF*α mRNA expressions were lower in abundance in cotyledon, caruncle, and uterus of SCPT ewes compared to healthy ewes (Figure 1; *P* < 0.05). The *sFlt1, KDR, eNOS, HIF1a*, and *HIF2a* mRNA expressions were greater in abundance in cotyledon, caruncle, and uterus of SCPT ewes compared to healthy ewes (*P* < 0.05). The *HIF2b* mRNA expression was not different between healthy and SCPT ewes (*P* > 0.05) in cotyledon, caruncle, and uterus. The *IL-8* mRNA expression was lower in abundance in cotyledon and in uterus (*P* < 0.05) but not in caruncle (*P* > 0.1) of SCPT ewes compared to healthy ewes. The *AdipoR1* mRNA expression was lower in abundance in both units of the placenta (*P* < 0.05) but not in the uterus (*P* > 0.1) of SCPT ewes compared to healthy ewes. The *IL6* mRNA expression was lower in abundance in the uterus of SCPT ewes compared to healthy ewes (*P* < 0.05) and the expression was not different in both units of placenta of SCPT and healthy ewes (*P* > 0.1).

### Morphometry Analysis of Placental Unit

**Figure 2:**
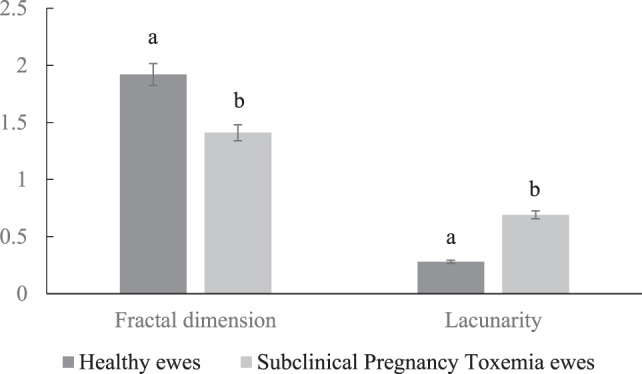
**Mean ± SEM fractal dimension and lacunarity of placental vascular network of healthy and subclinical pregnancy toxemic ewes**. Bars with different letters indicates parameters were significantly (*P* < 0.05) different between healthy and SCPT ewes.

The healthy ewes had increased fractal dimension and decreased lacunarity in their placental vascular network compared to SCPT ewes (Figure 2; *P* < 0.05).

### Predicted Functional Gene and miRNA Partners

**Figure 3:**
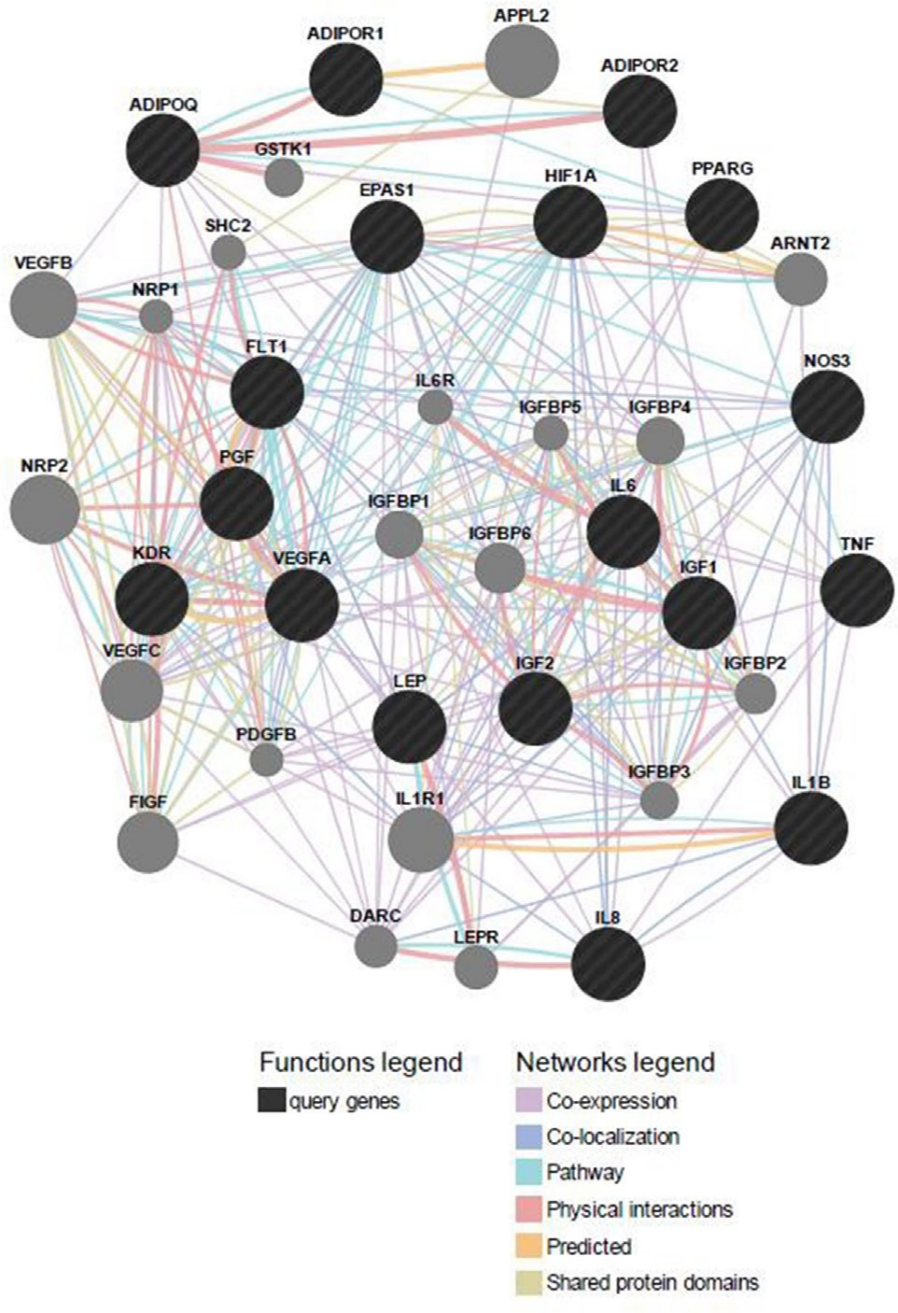
**Predicted functional gene partners: investigated genes were used as input genes; additional functional partners were obtained; and genes were connected by various links based on different functions**.

**Figure 4:**
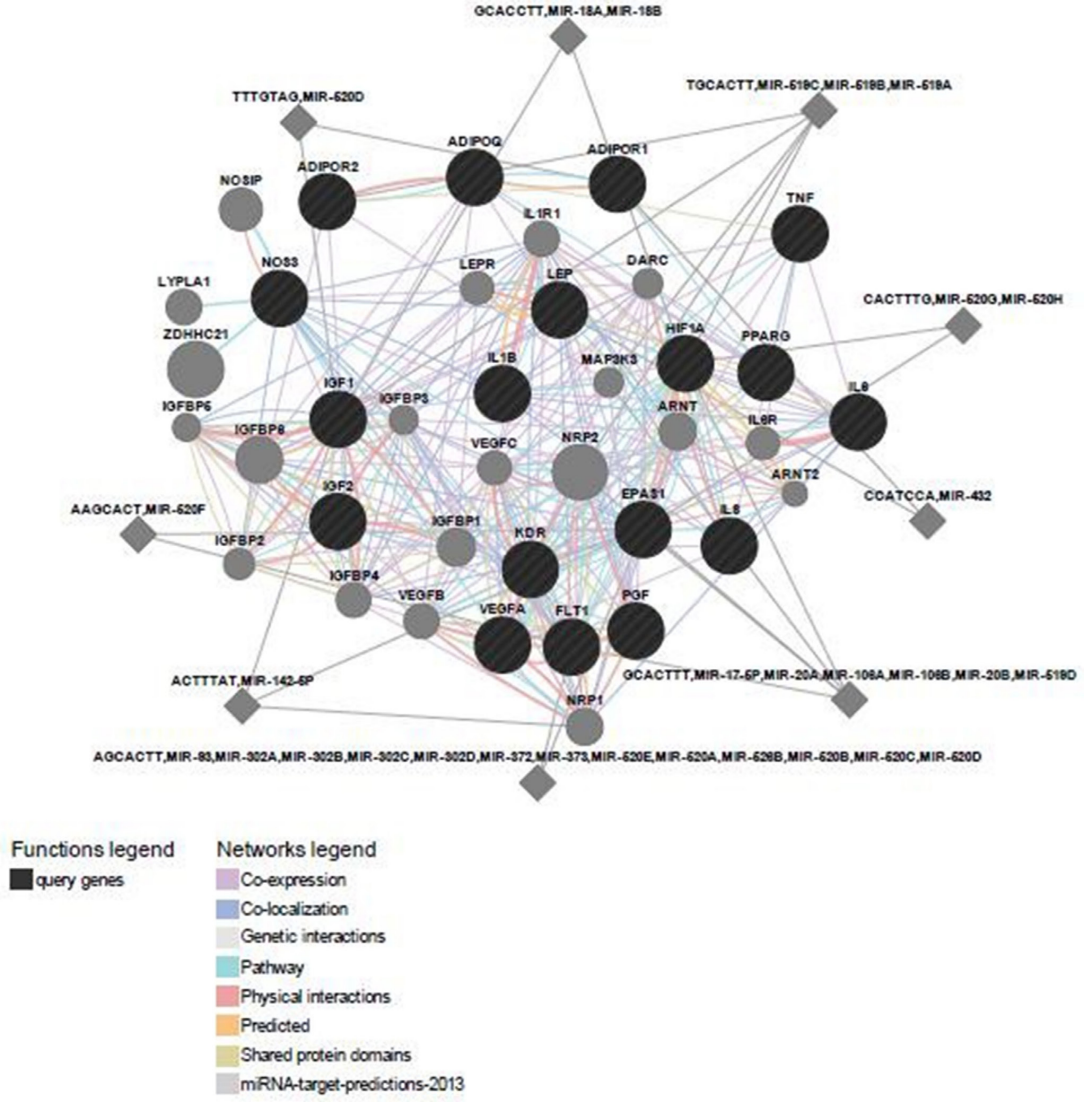
**Predicted functional gene and their miRNA partners**.

Predicted functional genes investigated in this study are interrelated (Figure 3). Different line colors represent the types of evidence for the association. Predicted association was made based on neighborhood, gene fusion, co-occurrence, coexpression, experiments, databases, text mining, homology, and at a confidence score of 0.90. The relationship network of functional genes and miRNA is presented in Figure 4.

**Figure 5:**
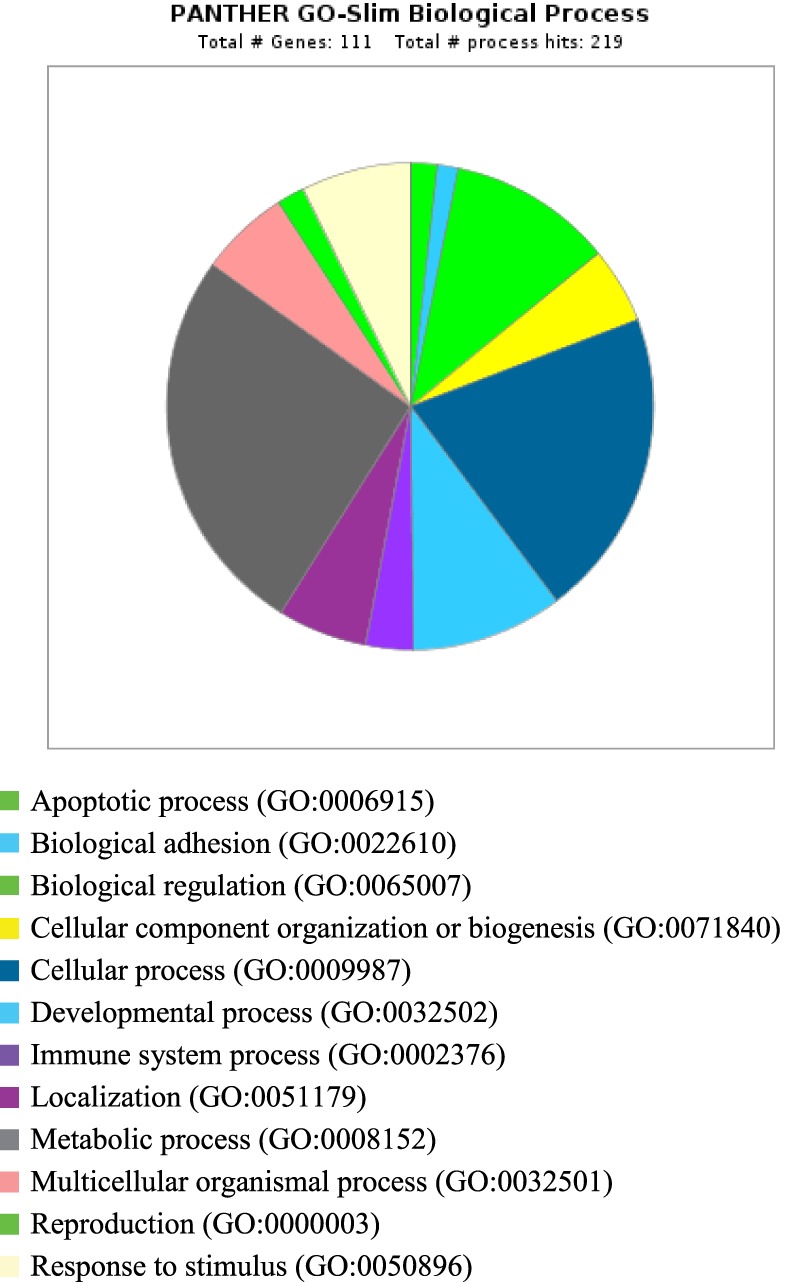
**Biological process for integrated genes and miRNAs in pregnancy toxemia in ewes**. GO biological process; total number of genes: 111; total number of process hit: 219.

**Figure 6:**
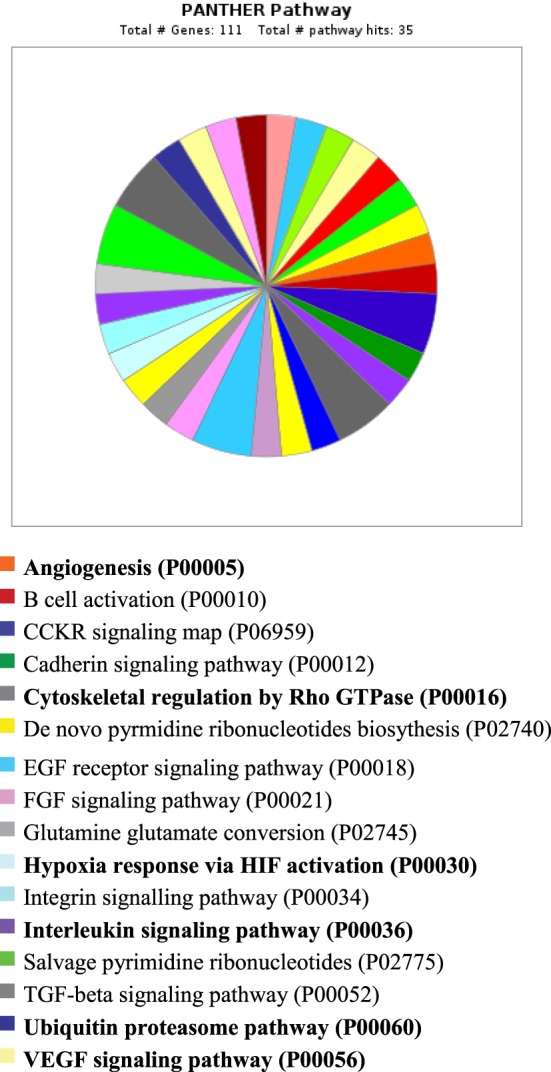
**Pathway for integrated miRNAs and genes in pregnancy toxemia in ewes**. PANTHER pathway; total number of genes: 111; total number of pathway hit: 35.

PANTHER analysis revealed that the predicted miRNA and associated target genes contributed to several biological processes, including apoptosis, biological adhesion, biological regulation, cellular component organization or biogenesis, cellular process, developmental process, immune system process, localization, metabolic process, multicellular organismal process, reproduction, and response to stimulus (Figure 5). The target genes were involved in several pathways including angiogenesis, cytoskeletal regulation, hypoxia response *via* HIF activation, interleukin signaling, ubiquitin proteasome, and VEGF signaling pathway (Figure 6).

## Discussion

The goal of this study was to elucidate the differences in gene expressions of the uteroplacental compartment and morphometry of placental vascular network between healthy and SCPT ewes. We used BCS and biochemical parameters to distinguish SCPT ewes from healthy ewes. The SCPT ewes had lowered BCS and blood glucose concentration and increased BHBA concentration without any clinical signs of the disease (4). The findings of the study indicated that expressions of the genes (*VEGF* and *PlGF*) associated to vascular remodeling were lower in abundances in SGPT ewes; the genes (*HIF1a, HIF2a*, and *eNOS*) associated to hypoxic condition were greater in abundances in SGPT ewes; morphometry analysis of angiogenic parameters of uteroplacental unit displayed reduced vascularization suggestive of hypoxic conditions in SCPT ewes compared to healthy ewes.

In this study, the BCS and concentrations of glucose were lower and BHBA was greater in SCPT ewes compared to healthy ewes. Susceptible, thin ewes develop ketosis because a chronically inadequate ration is offered or because other diseases such as lameness or dental diseases limit intake and, with increasingly insufficient energy to meet increasing fetal demands, the ewe or doe mobilizes more body fat, with resultant ketone body production and hepatic lipidosis. The ewes included in the study were observed to have normal appetite. Ewes with a poor BCS or that are overconditioned and carrying more than one fetus are most at risk of developing pregnancy toxemia, although the condition can occur even in ideally conditioned ewes on an adequate ration. In this study, three animals had twins and no differences between ewes carrying singletons and twins were detected.

A multifaceted variety of angiogenic growth factors are recognized as regulators of the vascularization process, and these include *VEGF, PlGF, sFlt-1, KDR*, and *HIF*s (30–32). The sheep placenta produces these angiogenic factors throughout gestation and tissue- and cell-specific patterns of expression have been documented in normal pregnancies (33) and in those where placental insufficiency and fetal growth restriction have been induced by maternal hyperthermia (34). In the current study, *VEGF* and *PlGF* gene expressions were lower in abundances, whereas *sFlt1* and *KDR* expression were greater in abundances in placenta and uterus of SCPT ewes compared to healthy ewes. In addition, *HIF1a, HIF2a*, and *eNOS* expressions were greater in abundance in placenta and uterus of SCPT ewes. In women, *sFlt-1* is increased, and *VEGF* and *PlGF* are decreased in placenta under hypoxic conditions (35–40). It is possible that the binding of *sFlt1* to free *VEGF* and *PlGF* reduces their availability, thereby causing endothelial cell dysfunction, leading to ischemia, proteinuria, and other maternal systemic symptoms in SCPT ewes (41, 42).

Hypoxia-inducible factor is the main regulator of the cellular response to low oxygen levels in mammalian species. The *HIF1a* and *HIF2a* both play important roles in vascular development and endothelial function (43–45). The *HIF1a* is the molecular link between hypoxia and pregnancy disorders as it induces antiangiogenic factor *sFlt1* and vasoconstrictor Urotensin-II (46, 47). The *HIF2a* reported to have effects on cell proliferation, invasion, and angiogenesis, processes important in placentation (48). So, in human, it is apparent that balanced expressions of HIFs are needed for normal vascularization, whereas excessive *HIF* results in endothelial dysfunction. In the present study, increased expressions of *HIF1a* and *HIF2a* in the placenta and the uterus of SCPT ewes plausibly induced excessive hypoxia and caused poor placental vascularization by suppressing *VEGF* and *PlGF via* increased *sFlt1* and *KDR*.

It is evident that *PPAR*γ plays a predominant role in normal vascular function (49, 50) and in the differentiation of labyrinthine trophoblast lineages (51) which, along with the fetal endothelium, form the vascular exchange interface with maternal blood (52). The *PPAR*γ null placentas develop a malformed labyrinth zone (52), suggesting a critical role for *PPAR*γ in the progression of normal pregnancy. Moreover, administration of a *PPAR*γ agonist improved several signs of this condition. Conversely, *PPAR*γ antagonist treatment to pregnant rats resulted in significant decrease in *VEGF* and significant increase in *sFlt-1*. In this study, *PPAR*γ mRNA abundances were lower in SCPT ewes compared to healthy ewes. This may have caused ischemia, endothelial dysfunction, proteinuria, and an imbalance of angiogenic proteins in SCPT ewes (41, 42, 53).

Pregnancy toxemia is characterized by a clustering of biochemical and clinical characteristics, including insulin resistance (17). Similar to other pregnancy complications, alterations in the levels of insulin, IGF1, leptin and adiponectin, cytokines, and VEGF also occur in this condition. Further, these metabolic syndromes are associated with a low-grade, chronic state of inflammation characterized by increased circulating free fatty acids, and chemoattraction of macrophages, which also produce inflammatory mediators into the local milieu (54–56). These effects are further amplified by the release of inflammatory cytokines, such as *IL1*β, *IL6*, and *TNF*α. It should be noted that adiponectin was implicated in the pathogenesis of insulin resistance. Administration of adiponectin significantly ameliorates insulin resistance. Further adiponectin enhances fatty acid oxidation and glucose uptake by decreasing circulating free fatty acids and improving whole-body insulin action (57). In this study, SCPT ewes had lower adiponectin expression in placenta and uterus plausibly lowered the glucose utilization by the uteroplacental unit contributed to hypoxic conditions. It should be noted that there is a functional network involving *VEGF, IGF*, and *MMP* in placenta and uterus, which is important for normal placentation (58). The *IGF2* signaling has been found to upregulate *VEGF* function. *IGF2* has effects on cell proliferation and apoptosis; lower levels of *IGF2* may conceivably reduce cell proliferation and placental mass, in addition to increasing apoptosis (59).

In this study, circulating isoprostane was increased in SCPT ewes compared to healthy ewes. Increased isoprostane concentrations are reported in normal and IUGR pregnancies (60, 61). It should be noted that mild oxidative stress and resultant increase in isoprostane might be involved in normal pregnancy. However, in placental abnormalities caused by oxidative stress, there is increased isoprostane generation (62) as noted in this study.

Remarkably, the genes investigated and predicted miRNAs and targeted genes were related to several biological functions. The targeted genes contributed to several biological processes, such as apoptotic process, biological adhesion, biological regulation, cellular component organization or biogenesis, cellular process, developmental process, immune system process, localization, metabolic process, multicellular organismal process, reproduction, and response to stimulus. The target genes were involved in several pathways including angiogenesis, cytoskeletal regulation, hypoxia response *via HIF* activation, interleukin signaling, ubiquitin proteasome, and *VEGF* signaling pathway. The biological functions explained the association of pregnancy toxemia (regulation of metabolic process: carbohydrate and glucose metabolism and catabolic process), placental vascularization (regulation of angiogenesis: blood vessel development, exocytosis and apoptosis and involvement of interleukins, endothelial growth factors, insulin-like growth factors, and adipokines), and hypoxic condition (regulation of nitric oxide synthase and hypoxia).

## Conclusion

Pregnancy is a dominant physiological state during which an alteration in metabolism may be expected because of a greater demand for nutrients by developing fetus. In addition to alterations in nutrient partitioning, the placentogenesis are tightly associated with diverse pathophysiological changes in the feto-maternal compartment. The findings of the study indicated that expressions of the genes associated with vascular remodeling were lower in abundances and that the genes associated with hypoxic condition were greater in abundances in the uteroplacental compartment in SCPT ewes. In addition, morphometry analysis of angiogenic parameters of uteroplacental unit displayed reduced vascularization suggestive of hypoxic conditions in SCPT ewes compared to healthy ewes. It is obvious that the factors that influence placental vascular development and angiogenesis as noted in this study set the course for hemodynamic changes and hence have a major impact on the rate of transplacental nutrient exchange, fetal growth, and health of the dam.

## Author Contributions

Acquisition of funding; conception, design, and collection of data; morphometry analysis; data analysis and interpretation; and drafting of the manuscript, tables, and figures. The author confirms being the sole contributor of this work and approved it for publication.

## Conflict of Interest Statement

The author declares that the research was conducted in the absence of any commercial or financial relationships that could be construed as a potential conflict of interest.
