# Supplementary material for: Subclinical Pregnancy Toxemia-Induced Gene Expression Changes in Ovine Placenta and Uterus
Source: Front Vet Sci. 2016 Aug 30;3:69. doi: 10.3389/fvets.2016.00069 (PMC5003868; doi:10.3389/fvets.2016.00069)
Supplement: Supplementary file 1 [file Table_1.DOCX]

Table 1. Primers used for cDNA amplification of the targets by RT-PCR

| Gene | Primer | Seq 5′ to 3′ | Accession number |
| --- | --- | --- | --- |
| *VEGF* | Forward | CCTCACCAAAGCCAGCACAT | AF071015.1 |
|  | Reverse | CGTCTGCGGATCTTGTACAAAC |  |
| *KDR* | Forward | GATGCTCGCCTCCCTTTGA | AF233076 |
|  | Reverse | GATCCCCATGCCAGCAATC |  |
| *SFlt1* | Forward | GCCACGCCTGAAATCTACCA | AF233077 |
|  | Reverse | GGCGTTGAGCGGAATGTAGT |  |
| *PlGF* | Forward | GCCGGTCATGAGGCTGT | NM_002632 |
|  | Reverse | GCAGTCACTGAAGAGTGTGAC |  |
| *eNOS* | Forward | TGGGCCGCATCCAGTG | NM_001129901 |
|  | Reverse | GAACATCTCCTGTGCTGAGCTG |  |
| *HIF1a* | Forward | TCAGCTATTTGCTGTGAGG | EU340260 |
|  | Reverse | TTCACAAATCAGCACCAAGC |  |
| *HIF1b* | Forward | AGA TGC AGG AAT GGA CTT GG | EU340261 |
|  | Reverse | CCT GGC CTT TTA ACT TCA CG |  |
| *HIF2a* | Forward | AAGTCAGCCACCTGGAAGG | EU340264 |
|  | Reverse | TCACACACATCATGCACTGG |  |
| *HIF2b* | Forward | AGGATGAGGTGTGGAAATGC | EU340265 |
|  | Reverse | CCTCAGAGTGGCAGAACTCC |  |
| *AdipoQ* | Forward | ATCAAACTCTGGAACCTCCTATCTAC | NM_174742 |
|  | Reverse | TTGCATTGCAGGCTCAAG |  |
| *AdipoR1* | Forward | GCTCCCGCTAGCAACAGGGC | NM_001034055 |
|  | Reverse | CCTGCAGGGGCAGTGTGAGC |  |
| *AdipoR2* | Forward | CCCAGTACCGGGGCGTGAGA | NM_001040499 |
|  | Reverse | GGAAGTGGACGAACGCCCA |  |
| *PPARγ* | Forward | ATGTCTCATAATGCCATCAGGTT | AY179866 |
|  | Reverse | GATAACAAACGGTGATTTGTCTGTC |  |
| *IGF1* | Forward | CCTGGATTTCTTTTTGCCTCAT | NM_001009774.2 |
|  | Reverse | GCTGAAGGCGAGCAAGCA |  |
| *IGF2* | Forward | CTGGTGGACACCCTCCAGTT | NM_001009311.1 |
|  | Reverse | TCCGGAAGCACGGTCGTA |  |
| *LEP* | Forward | CCACTCACCAGCATGCAAAG | NM_173928 |
|  | Reverse | TTCAAAGGGATGTGGCATCA |  |
| *IL-1b* | Forward | TCACAGGAAATGAGCCGAGAA | NM_001009465 |
|  | Reverse | CAGCTGCAGGGTCGGTGTAT |  |
| *IL-6* | Forward | ACACCACCCCAAGCAGACTACT | NM_001009392 |
|  | Reverse | CCCAGATTGGAAGCATCCAT |  |
| *IL-8* | Forward | GCCAGAAGAAACCTGACAAAAAG | NM_001009401 |
|  | Reverse | GCAGTGTGGCCCACTCTCA |  |
| *TNFα* | Forward | GACCCTCCTCATCCCCTTCT | [NM_001024860.1](http://www.ncbi.nlm.nih.gov/entrez/viewer.fcgi?db=nucleotide&id=67845988) |
|  | Reverse | AGCCCACCCATGTCAAGTTC |  |
| *β-actin* | Forward | CCAAGGCCAACCGTGAGA | NM_001009784.1 |
|  | Reverse | AGCCTGGATGGCCACGT |  |
