# Supplementary material for: Subclinical Pregnancy Toxemia-Induced Gene Expression Changes in Ovine Placenta and Uterus
Source: Front Vet Sci. 2016 Aug 30;3:69. doi: 10.3389/fvets.2016.00069 (PMC5003868; doi:10.3389/fvets.2016.00069)
Supplement: Supplementary file 2 [file Table_2.DOCX]

Table 2. Mean ± SEM alpha tocopherol and gamma tocopherol concentrations in placenta, uterus and serum of healthy ewes (n = 5) and ewes with subclinical pregnancy toxemia (n = 5).

| Sample | Group | aT (µg/kg) | gT (µg/kg) |
| --- | --- | --- | --- |
| Placenta | Healthy ewes | 3.48 ± 0.17 | 51.1 ± 10.28 |
|  | SCPT ewes | 3.34 ± 0.18 | 58.9 ± 12.96 |
| Uterus | Healthy ewes | 2.85 ± 0.15 | 126.7 ± 27.87 |
|  | SCPT ewes | 2.74 ± 0.18 | 145.8 ± 29.16 |
| Serum | Healthy ewes | 6.26 ± 0.31 | 552.7 ± 66.32 |
|  | SCPT ewes | 5.98 ± 0.36 | 498.4 ± 74.76 |

aT- alpha tocopherol; gT-gamma tocopherol; SCPT, subclinical pregnancy toxemia; Placenta –Caruncle and cotyledon.
