# Supplementary material for: Subclinical Pregnancy Toxemia-Induced Gene Expression Changes in Ovine Placenta and Uterus
Source: Front Vet Sci. 2016 Aug 30;3:69. doi: 10.3389/fvets.2016.00069 (PMC5003868; doi:10.3389/fvets.2016.00069)
Supplement: Supplementary file 3 [file Table_3.DOCX]

Table 3: Top ranked (based on total target score of miRDB) targeted genes for miRNAs

| **microRNA ID** | **Targeted genes** |
| --- | --- |
| miR-17-5p | *ZNF800, ARID4B, ADARB1, PTPN4, PKD2, GAB1, SLC40A1, ZNFX1, FBXL5, EPHA5* |
| miR-18A | *NEDD9, BBX, ZBTB47, PHF19, RORA, INADL, MAP7D1, ERI1,* ***HIF1A,*** *CDK19* |
| miR-18B | *NEDD9, RORA, BBX, MAP7D1, INADL, PHF19, ZBTB47, CDK19, ERI1, DICER1* |
| miR-20A | *KATNAL1, ARID4B, PKD2, SLC40A1, PDCD1LG2, ZNF800, PTPN4, ZNFX1, FBXL5, EPHA4* |
| miR-20B | *ZNFX1, PTPN4, PDCD1LG2, ADARB1, PKD2, ZNF800, ARID4B, FBXL5, SLC40A1, KATNAL1* |
| miR-93 | *PDCD1LG2, ZNFX1, FBXL5, EPHA4, ZNF800, PKD2, SLC40A1, MAP3K2, EZH1, SACS* |
| miR-106A | *ZNF800, ARID4B, ADARB1, PTPN4, PKD2, GAB1, SLC40A1, ZNFX1, FBXL5, EPHA4* |
| miR-106B | *PTPN4, ARID4B, EPHA4, PKD2, PDCD1LG2, SLC40A1, FBXL5, ZNF800, ADARB1 , ZNFX1* |
| miR-142-5p | *ZFPM2, BAI3, AFF4, DIAPH2, AHR, ARID4B, SLC18A2, C11orf87, C8orf34, PHYHIPL* |
| miR302A | *YOD1, OXR1, LATS2, NR2C2, ZNF800, CROT, CYBRD1, ZNF367, REEP3, RSBN1* |
| miR-302B | *YOD1, OXR1, LATS2, NR2C2, ZNF800, CROT, CYBRD1, ZNF367, REEP3, RSBN1* |
| miR-302C | *OXR1, YOD1, NR2C2, CROT, ZNF800, LATS2, CYBRD1, MPC1, ZNRF3, REEP3* |
| miR-302D | *OXR1, LATS2, CROT, YOD1, CYBRD1, NR2C2, ZNF800, ZNF367, RSBN1, PPP6C* |
| miR-372 | *LATS2, NR2C2, CYBRD1, YOD1, ZNF800, OXR1, CROT, REEP3, ZNRF3, ZNF367* |
| miR-373 | *OXR1, YOD1, CROT, ZNF800, CYBRD1, NR2C2, LATS2, RSBN1, REEP3, ZNF367* |
| miR-432 | *CELSR2, MAN1A2, COL4A5, ZMIZ1, ARHGEF4, FAM178A, PLK3, BACE1, TBC1D22B, UCK2* |
| miR-519A | *ARID4B, FBXL5, SLAIN1, ARHGAP24, HEG1, TGFBR2, KIF13A, PDE3B, LRIG1, KDM2A* |
| miR-519B | *ARID4B, FBXL5, SLAIN1, ARHGAP24, HEG1, TGFBR2, KIF13A, PDE3B, LRIG1, KDM2A* |
| miR-519C | *FBXL5, SLAIN1, TGFBR2, ARHGAP24, KIF13A, ARID4B, HEG1, CMPK1, CPEB1, MTMR4* |
| miR-519D | *PDCD1LG2, PKD2, ZNF800, EPHA4, FBXL5, ZNFX1, SLC40A1, ANKRD29, ARID4A, PXK* |
| miR-520A | *YOD1, OXR1, NR2C2, ZNF800, CYBRD1, LATS2, CROT, MPC1, ZNF367, ZNRF3* |
| miR-520B | *ZNF800, RSBN1, YOD1, LATS2, CYBRD1, CROT, OXR1, NR2C2, ZNF367, MPC1* |
| miR-520C | *ZNF800, RSBN1, YOD1, LATS2, CYBRD1, CROT, OXR1, NR2C2, ZNF367, MPC1* |
| miR-520D | *FBXO30, ELAVL2, PUM2, SMURF2, RBM39, SEH1L, EGFL6, IGSF3, LRRTM3, GLUD1* |
| miR-520E | *CYBRD1, NR2C2, OXR1, ZNF800, YOD1, LATS2, CROT, ZNF367, MPC1, RSBN1* |
| miR-520F | *C2orf69, CROT, FYCO1, DDHD1, IL6ST, PRRX1, ARID4B, NOVA1, ARMC8, RALGPS2* |
| miR-520G | *SLC35A5, SMOC2, RRAGD, HIPK3, ZFYVE20, HIF1A, TNRC6A, JAZF1, LIMK1, ABHD2* |
| miR-520H | *SLC35A5, SMOC2, ZFYVE20, JAZF1, HIF1A, RRAGD, HIPK3, TNRC6A, KLHL29, VLDLR* |
| miR-526B | *PANK3, PDIA6, RIMKLB, FKBP5, FGD4, SPTSSA, ZFHX4, CHST15, HMG20A, DTX3L* |
